# Involvement of a G Protein Regulatory Circuit in Alternative Oxidase Production in *Neurospora crassa*

**DOI:** 10.1534/g3.119.400522

**Published:** 2019-08-23

**Authors:** Natasa Bosnjak, Kristina M. Smith, Iman Asaria, Adrian Lahola-Chomiak, Nishka Kishore, Andrea T. Todd, Michael Freitag, Frank E. Nargang

**Affiliations:** * Department of Biological Sciences, University of Alberta, Edmonton, Alberta, Canada T6G 2E9 and; † Department of Biochemistry and Biophysics, Oregon State University, Corvallis, Oregon 97331-4003

**Keywords:** alternative oxidase, AOX, *Neurospora crassa*, G protein, *flbA*

## Abstract

The *Neurospora crassa* nuclear *aod-1* gene encodes an alternative oxidase that functions in mitochondria. The enzyme provides a branch from the standard electron transport chain by transferring electrons directly from ubiquinol to oxygen. In standard laboratory strains, *aod-1* is transcribed at very low levels under normal growth conditions. However, if the standard electron transport chain is disrupted, a*od-1* mRNA expression is induced and the AOD1 protein is produced. We previously identified a strain of *N. crassa*, that produces high levels of *aod-1* transcript under non-inducing conditions. Here we have crossed this strain to a standard lab strain and determined the genomic sequences of the parents and several progeny. Analysis of the sequence data and the levels of *aod-1* mRNA in uninduced cultures revealed that a frameshift mutation in the *flbA* gene results in the high uninduced expression of *aod-1*. The *flbA* gene encodes a regulator of G protein signaling that decreases the activity of the Gα subunit of heterotrimeric G proteins. Our data suggest that strains with a functional *flbA* gene prevent uninduced expression of *aod-1* by inactivating a G protein signaling pathway, and that this pathway is activated in cells grown under conditions that induce *aod-1*. Induced cells with a deletion of the gene encoding the Gα protein still have a partial increase in *aod-1* mRNA levels, suggesting a second pathway for inducing transcription of the gene in *N. crassa*. We also present evidence that a translational control mechanism prevents production of AOD1 protein in uninduced cultures.

Mitochondria contain proteins encoded by both nuclear DNA and mtDNA. To achieve proper mitochondrial biogenesis, communication between the mitochondria and the nucleus is required to coordinate the expression of genes found in the two organelles. Since the vast majority of genes encoding mitochondrial proteins are found in the nucleus, changes in the functional status of mitochondria must be communicated to the nucleus to modulate the expression of nuclear-encoded mitochondrial proteins according to specific conditions. Communication from the mitochondria to the nucleus has been termed retrograde regulation and has been shown to occur in many organisms (Liu and Butow 2006; Jazwinski 2013; da Cunha *et al.* 2015; Cardamone *et al.* 2018; Guaragnella *et al.* 2018; Isaac *et al.* 2018).

The alternative oxidase (AOX) is a mitochondrial enzyme that is encoded in the nucleus. AOX transfers electrons from ubiquinol directly to molecular oxygen, thus bypassing complexes III and IV of the standard electron transport chain (Vanlerberghe and McIntosh 1997; Joseph-Horne *et al.* 2001; McDonald 2008; Nargang and Kennell 2010; Vanlerberghe 2013). It is an interfacial membrane protein that localizes to the inner leaflet of the mitochondrial inner membrane (Moore *et al.* 2013; Shiba *et al.* 2013; May *et al.* 2017). AOX is present in a wide range of species although it has not been identified in any higher animal (McDonald and Vanlerberghe 2006; McDonald 2008; McDonald 2009; Neimanis *et al.* 2013). In many organisms a variety of factors that affect mitochondrial function can induce transcription of the gene to high levels (Vanlerberghe and McIntosh 1997; Joseph-Horne *et al.* 2001; McDonald 2008; Nargang and Kennell 2010; Vanlerberghe 2013). Thus, AOX serves as an excellent system for the study of retrograde regulation since disruption of mitochondrial function results in increased expression of the nuclear gene encoding AOX.

We have studied the regulation of AOX production in the filamentous fungus *Neurospora crassa*. The organism contains two genes encoding AOX called *aod-1* and *aod-3*, although no conditions have been found that result in expression of *aod-3* (Tanton *et al.* 2003). In standard laboratory strains the *aod-1* gene is transcribed at very low levels when the organism is grown in normal growth media. However, induction of *aod-1* transcription and translation of the mRNA occurs when cells are grown in the presence of inhibitors that affect the function of the standard mitochondrial electron transport chain, such as antimycin A or cyanide (Lambowitz and Slayman 1971; Henry and Nyns 1975; Bertrand *et al.* 1983; Lambowitz *et al.* 1989; Tanton *et al.* 2003). The expression of *aod-1* and production of the AOD1 protein are also increased in strains carrying mutations that affect standard electron transport chain function (Tissieres *et al.* 1953; Lambowitz *et al.* 1972; Bertrand *et al.* 1983; Li *et al.* 1996). Growth in the presence of chloramphenicol (Cm) also increases *aod-1* expression since it indirectly affects the standard electron transport chain by inhibiting mitochondrial (but not cytosolic) protein synthesis, thus reducing the levels of standard electron transport chain complexes that contain mitochondrial-encoded subunits (Lambowitz and Slayman 1971; Nargang and Kennell 2010).

In *N. crassa*, several genes required for *aod-1* expression have been identified (Bertrand *et al.* 1983; Descheneau *et al.* 2005; Nargang *et al.* 2012). The best studied of these are called *aod-2* and *aod-5*. Gene rescue experiments, sequencing, and bioinformatics revealed that *aod-2* and *aod-5* encode zinc cluster transcription factors (Chae *et al.* 2007b). These factors bind constitutively as a heterodimer to an alternative oxidase induction motif (AIM) that exists in the promoter region of the *aod-1* gene (Chae *et al.* 2007a; Qi *et al.* 2017). Transcription of *aod-1* is induced in response to an unknown signal(s) generated when the standard electron transport chain is disrupted. In the absence of either *aod-2* or *aod-5*, the induction of *aod-1* is essentially eliminated (Tanton *et al.* 2003; Chae *et al.* 2007a; Chae *et al.* 2007b; Chae and Nargang 2009; Qi *et al.* 2017). Orthologs of the AOD2 and AOD5 proteins exist in other fungi and have been shown to be required for AOX induction in *Aspergillus nidulans* (Suzuki *et al.* 2012) and *Podospora anserina* (Bovier *et al.* 2014). Thus, the AOD2 and AOD5 proteins are directly involved in control of *aod-1* expression.

However, there is evidence that additional factors play a role in *aod-1* expression. Several other genes were identified as being required for full *aod-1* expression during an examination of the *N. crassa* single gene deletion library (Colot *et al.* 2006; Nargang *et al.* 2012) and in a genetic screen for regulatory mutants unable to induce the enzyme (Descheneau *et al.* 2005). Furthermore, during the development of a reporter strain for use in the screen for regulatory mutants, we serendipitously identified a *N. crassa* strain (called T1P11) carrying a mutant tyrosinase gene that consistently had high levels of *aod-1* mRNA in non-inducing conditions, though no AOD1 protein was produced (Descheneau *et al.* 2005). Although the tyrosinase gene is required for the synthesis of melanin (Kupper *et al.* 1989) it was conceivable that loss of the gene might affect *aod-1* transcript levels. To determine if the tyrosinase mutation was responsible for the upregulation, a cross between T1P11 and a wild-type strain with low levels of uninduced transcript (NCN233) was performed. Analysis of the progeny revealed that the tyrosinase mutation and the high levels of uninduced *aod-1* transcripts segregated independently (Descheneau *et al.* 2005). In the present work we have confirmed this result and have shown that other genes known to affect *aod-1* expression are not altered in T1P11. To identify the gene(s) causing the effect, we determined the genomic sequence of T1P11, NCN233, and progeny from a cross between these two strains. Our analysis revealed that a frameshift mutation in a the *flbA* gene, which encodes a Regulator of G protein Signaling (RGS protein), is responsible for the increased expression of *aod-1* in non-inducing conditions.

## Materials and Methods

### Handling and growth of N. crassa strains

Table 1 lists the strains used in this study. Unless otherwise stated, all handling and growth of *N. crassa* strains was conducted as previously described (Davis and De Serres 1970). Briefly, conidia for growing liquid cultures were produced by inoculating conidia in the center of 250 ml or 125 ml Erlenmeyer flasks containing 50 or 25 ml, respectively, of Vogel’s salts, 1.5% sucrose, trace elements, and biotin (VSuTB). Medium was solidified with 1.5% agar. Any additional supplements were added as required. The most commonly used supplement was pantothenic acid at a final concentration of 10 mg/liter. Flasks were incubated for two to three days at 30° and then placed at room temperature to allow conidiation to occur in a well-lit room for four to five days. Conidia were used up to 10 days after their formation. Stocks were maintained on agar slants containing VSuTB, plus any required supplements, and kept frozen at −80°.

**Table 1:** Strains used in this study

Strain	Origin	Genotype	Uninduced *aod-1* mRNA level
**NCN233**	Nargang lab	*pan-1*, *A*	low
**T1P11**	S. Free (U. Buffalo)	*T*^-^, *al-2*, *a*	high
**3**	NCN233 x T1P11	*T^+^*, *al-2^+^*, *pan-1*	low
**4**	NCN233 x T1P11	*T^+^*, *al-2^+^*, *pan-1*	low
**6**	NCN233 x T1P11	*T*^-^, *al-2*, *pan-1*	low
**9**	NCN233 x T1P11	*T*^-^, *al-2*, *pan-1^+^*	high
**16**	NCN233 x T1P11	*T*^-^, *al-2*, *pan-1^+^*	high
**19**	NCN233 x T1P11	*T^+^*, *al-2^+^*, *pan-1^+^*	high
**23**	NCN233 x T1P11	*T*^-^, *al-2*, *pan-1^+^*	high
**26**	NCN233 x T1P11	*T*^-^, *al-2*, *pan-1*	high
**33**	NCN233 x T1P11	*T*^-^, *al-2^+^*, *pan-1*	low
**34**	NCN233 x T1P11	*T^+^*, *al-2^+^*, *pan-1*	low
**36**	NCN233 x T1P11	*T^+^*, *al-2^+^*, *pan-1^+^*	low
***∆flbA* (12372)***^a^*	FGSC*^b^* #12372	*∆flbA*, *a*	high
***∆flbA* (12373)**	FGSC #12373	*∆flbA*, *A*	high
***∆kin-9* (13484)**	FGSC #13484	*∆kin-9*, *a*	low
***∆kin-9* (13485)**	FGSC #13485	*∆kin-9*, *A*	low
***∆gna-1***	FGSC #12370	*∆gna-1*, *a*	low

a All strains containing a deletion of a specific gene were obtained from the *N. crassa* single gene deletion library (Colot *et al.* 2006).

b Fungal Genetics Stock Center.

Liquid cultures were grown by collecting conidia from flasks containing agar solidified VSuTB using sterile distilled H_2_O. Conidia were counted using a hemocytometer, and added to VSuTB liquid medium at a concentration of 10^6^ conidia/ml, followed by shaking at 30° for 12 to 18 hr, or as indicated. When required, Cm (at a final concentration of 2 mg/ml) was added to media as an inducer of *aod-1* transcription and production of the AOD1 protein.

### Quantitative real time polymerase chain reaction (qPCR)

To isolate RNA, conidia were grown in liquid cultures for 12-14 hr in medium lacking Cm (-Cm) or 16-18 hr in medium containing Cm (+Cm). Mycelial pads were obtained via vacuum filtration. A portion of the pad weighing approximately 100 mg fresh weight was flash frozen in liquid nitrogen. All equipment (glassware, Buchner funnels, forceps, mortars, pestles, and aluminum foil) was baked at 240° for at least 24 hr to eliminate RNase contamination. RNA was isolated using the Qiagen RNeasy Plant Kit following the manufacturer’s instructions for fungi (Qiagen Inc, Hilden, Germany). RNA concentrations were measured using a Nanodrop ND 1000 spectrophotometer (Thermo Scientific, Rockford) and RNA integrity was analyzed using the Bioanalyzer 2100 Nano chip (Agilent Technologies, Santa Clara). Only samples with RNA integrity numbers greater than 9.6 were used. cDNA was synthesized using 1 μg of RNA per reaction with Superscript III (Invitrogen, Carlsbad) and was diluted 20-fold with DNAse-free distilled H_2_O for use in qPCR. qPCR was performed using the StepOnePlus RT-PCR system (Applied Biosystems, Foster City). The reactions contained 2.5 μl of the 20-fold diluted cDNA, 2.5 μL diluted primer and 5 μl KAPA SYBR Fast qPCR master mix (KAPA Biosystems, USA) for a total volume of 10 μL per well on a 96-well plate. β-tubulin was used as an endogenous control for ΔΔCt analysis. Unless stated otherwise, all qPCR data are based on four biological replicates, each with three technical replicates. Statistical comparison between samples was done using the Students *t*-test in Microsoft Excel.

### Isolation of mitochondria

Cultures were grown in 250 ml of liquid VSuTB (with any needed supplements) for 16 hr without Cm or 18 hr in the presence of Cm (unless stated otherwise). Cultures were vacuum filtered to obtain mycelium pads. Pads were kept on ice until they could be processed. Mycelia were broken by grinding with sand and mitochondria were isolated using the buffers and differential centrifugation procedure described previously (Nargang and Rapaport 2007).

### Electrophoresis of proteins and western blots

Acrylamide gels for sodium dodecyl sulfate polyacrylamide gel electrophoresis (SDS-PAGE) were prepared as described by Sambrook and Russell (Sambrook and Russell 2001). The final acrylamide concentration was 12.5% with an acrylamide:bis-acrylamide ratio of 29:1. 30 μg of mitochondrial protein was loaded in one lane of a gel. Following electrophoresis proteins were transferred onto a Bio Rad Immun-Blot PVDF membrane (Bio Rad, Berkeley) as described (Towbin *et al.* 1979). Following transfer, the membrane was blocked for 1 hr at room temperature in blocking solution (5% fat-free skim milk powder in TBST (0.2 M Tris-HCl pH 7.5, 0.15 M NaCl, 0.5% Tween 20)) with gentle shaking. The blocking solution was removed and the membrane was incubated with gentle shaking for 1 hr with primary antibody at an appropriate dilution in blocking solution. The membrane was then washed three times, with gentle shaking for 5 min with TBST, followed by a 1 hr incubation with gentle shaking at room temperature with secondary antibody (1:3000 dilution of goat anti-rabbit antibody HRP-conjugate solution) in 50 ml blocking solution. The membrane was then washed three times for 10 min with TBST, and once with TBS (TBST without Tween 20). Detection of bound antibodies was facilitated using the LumiGLO chemiluminescent detection assay (KPL, Maryland) with Kodak XAR film (Eastman Kodak, New York).

### Generation of PCR products and isolation of DNA from gels

All PCR products were amplified using Phusion High Fidelity PCR (New England Biolabs, Ipswich). Primers were obtained from IDT (Integrated DNA Technologies, Coralville). Following PCR reactions, DNA was size-separated via gel electrophoresis on 1% agarose gels. Bands of interest were excised with a scalpel on a UV transilluminator, and DNA was extracted using a QiaQuick Gel extraction kit (Qiagen, Hilden, Germany), following the manufacturer’s instructions.

### Genomic DNA isolation

*N. crassa* genomic DNA was isolated using modifications of a previously described procedure (Wendland *et al.* 1996). Liquid cultures were grown in VSuTB at 30° for approximately 24 to 48 hr. Mycelial pads were obtained by vacuum filtration, and were stored on ice prior to processing. Approximately 2 g of mycelium was used per preparation. Mycelium was ground using a mortar and pestle on ice with 1.5 g sand and 5 ml New Isolation Buffer (100 mM Tris-HCl pH 8.0, 10 mM EDTA, 1% SDS). The mixture was transferred to a tube for the Sorvall SA-600 rotor (Sorvall, Mandel Scientific, Guelph) for later use in centrifugation. The mixture was then brought to 10 ml with New Isolation buffer, and covered with parafilm. The tubes were shaken vigorously, inverted slowly for approximately 1 min, then incubated in a 70° water bath for 1 hr. Tubes were then placed on ice for 10 min, after which 0.64 ml of 8 M potassium acetate pH 6.0, was added to each tube. The samples were left on ice for 1 hr. Tubes were placed in a Sorvall RC-5C Plus centrifuge with an SA-600 rotor (Sorvall, Mandel Scientific, Guelph) and centrifuged at 14 000 × g for 15 min. The supernatant was transferred to a clean SA-600 tube. An equal volume of isopropanol was added to the sample and mixed gently. The DNA precipitate was spooled around a sterile glass Pasteur pipette, transferred to a sterile disposable 15 ml tube, rinsed with 70% ethanol, and allowed to air dry. The pellet was then suspended in 400 μl 1 mM EDTA pH 8.0, and transferred to an Eppendorf tube. 200 μl HiSalt Buffer (100 mM NaCl, 25 mM Tris-base pH 7.4, 2 mM Na_2_EDTA) and 15 μl of previously boiled RNAse A solution (10 mg/ml) was added. The dissolved pellet was incubated at 37° for 30 min. An equal volume of phenol/chloroform (1:1) was then added. The tubes were shaken vigorously for 1 min and centrifuged for 1 min at maximum speed in a Sorvall Biofuge pico centrifuge (Sorvall, Mandel Scientific, Guelph). The top layer was transferred to a new Eppendorf tube and the phenol/chloroform extraction procedure was repeated once more. To precipitate final genomic DNA, one tenth volume of 3M sodium acetate, as well as three volumes of 95% ethanol were added to the Eppendorf tube and mixed. The tube was centrifuged at maximum speed for 5 min. The pellet was washed once more with 70% ethanol, centrifuged at maximum speed for 1 min, and the remaining ethanol was removed. The pellet was left to air dry for 1 hr at room temperature and was then resuspended in sterile distilled H_2_O.

### Sequencing of DNA fragments

DNA sequencing reactions were set up according to the specifications of the MBSU (Molecular Biology Services Unit) at the University of Alberta. DNA sequencing was conducted by the MBSU using a 3730 Analyzer (Applied Biosystems, Foster City). FinchTV (Geospiza Inc., Seattle) and NCBI BLAST (https://blast.ncbi.nlm.nih.gov/Blast.cgi) were used for sequence alignment and analysis.

### Whole Genome DNA Sequencing

Genomic DNAs from four strains (Table 1) expressing high uninduced levels of *aod-1* transcript (strains T1P11, 19, 23 and 26) and four expressing low levels of uninduced *aod-1* transcript (strains NCN233, 3, 4 and 6) were sequenced using the Illumina HiSeq 2000 platform (Illumina, San Diego) with a read length of 101 nucleotides. The sequences were mapped with the Burrows-Wheeler Aligner (BWA: http://bio-bwa.sourceforge.net/) (Li and Durbin 2009) to the wild type OR74A sequence (assembly 10) available at the FungiDB database (http://fungidb.org/fungidb/). The assembly 10 sequence is hereafter referred to as the reference sequence.

### Analysis of Whole Genome Sequencing

The genomic sequence data were mounted in g-browse (http://ascobase.cgrb.oregonstate.edu/cgi-bin/gb2/gbrowse/ncrassa_public/) (Stein *et al.* 2002) to allow viewing of each strain’s sequence relative to the reference sequence and to each other. Variants were called with the GATK HaplotypeCaller tool followed by the Joint Genotyper (GenotypeGVCFs) (https://software.broadinstitute.org/gatk/) to generate a vcf (variant call format) file. This file was opened in the IGV (Integrative Genomics Viewer) (Robinson *et al.* 2011; Thorvaldsdottir *et al.* 2013) which also allows viewing of each strain’s sequence relative to each other and the reference sequence. The vcf file was also exported to Microsoft Excel using the delimited column data format with “tab” and “space” as the delimiters. This resulted in an Excel table where changes present in each strain, relative to the reference sequence, were in separate columns. Other columns listed the supercontig, and numbered the position of each change (according to the reference sequence) and the actual sequence change. The sites of mutations occurring in any strain were listed in columns corresponding to each strain. In these columns, each cell contained a binary system where, at a specific position in the genome, a “0” indicated a match to the reference sequence in a given strain while a “1” indicated a change relative to the reference sequence for that strain.

In the Excel sheet, conditional formatting criteria was applied to columns corresponding to each strain to allow for filtering of the data. Conditional formatting involved the creation of a “New Rule” in the “Classic” style from the drop-down menu. Columns for all strains were formatted so that cells that began with “1” were designated a color, for example, pink. Cells that began with “0”, indicating positions that match the reference sequence, were kept white. This allowed a choice between seeing positions that matched the reference sequence (white) or positions that were different from the reference sequence (pink). Columns corresponding to the low *aod-1* expression strains (NCN233, 3, 4, and 6) were first filtered for white, indicating no change from the reference sequence at a certain position. Following this, high-expressing *aod-1* transcript strains (T1P11, 19, 23, 26) were analyzed on an individual basis. Each column corresponding to a high-expressing *aod-1* transcript strain was filtered for the color pink so as to select only variants. Variants fit the criteria of beginning with “1” and were designated a color. The positions of the variants were copied, and then pasted into another spreadsheet with a VBA (Visual Basics for Application) FindData code (explained below). Before moving onto a different high-expressing *aod-1* transcript strain, the variant color filter for the previous high-expressing strain was cleared. This was done so as to not select for variants that are present in different high expressing strains, allowing a full list of variants to be generated independently of other variants in other high-expressing strains. A filter for low-expressing strains remained at “white” for the entire filtering analysis in Excel to ensure that no changes present in high-expressing strains would also be present in low-expressing strains.

To analyze the positions of variants in each strain, a VBA code, which we named “FindData”, was written in a different Excel spreadsheet (Figure S1) and took the position of the change in question and searched through the list of known coding regions in the *N. crassa* genome in the supercontigs transcript annotation file (assembly 10) from the BROAD Institute. The filtering process described above resulted in a list of positions with changes in only high-expressing strains throughout the genome, including non-coding regions, coding regions, and UTRs. If the position was in the coding region of a protein coding gene, the program would list the NCU number of the gene. One chromosome was examined at a time to meet Excel’s RAM restrictions. A list of NCU numbers with changes in coding regions was generated for each strain expressing high levels of uninduced *aod-1* transcript (T1P11, 19, 23, and 26). The list of NCU numbers was then analyzed in the Venny web based software (Oliveros 2007-2015) to generate a Venn diagram, which enabled identification of genes that were mutated in all four high expressing strains.

### Analysis of protein sequences

Protein sequences were aligned using Clustal Omega (Goujon *et al.* 2010; Sievers *et al.* 2011). Identification of protein domains was done using the Inter-Pro protein sequence analysis and classification software at the European Bioinformatics Institute (GB) (https://www.ebi.ac.uk/interpro/) (McWilliam *et al.* 2013).

### Data availability

Strains and other materials used in this work are available upon request. Genomic sequence data are available at the NCBI SRA database, accession number PRJNA542366. Supplemental material available at FigShare: https://doi.org/10.25387/g3.9701600.

## Results

### Different levels of uninduced aod-1 mRNA in different strains

We will use the terms “inducing conditions” or “induced” to refer to the growth of cells in the presence of Cm (+Cm). Growth in Cm gives rise to elevated *aod-1* mRNA levels and results in the production of AOD1 protein in wild type cells. Growth in the absence of Cm (-Cm) or other inducers of *aod-1*, will be referred to as “non-inducing conditions” or “uninduced”. We previously reported (Descheneau *et al.* 2005) that a tyrosinase gene (gene symbol *T*, mutant allele *T*^-^, wild type allele *T^+^*) mutant strain named T1P11 (Fuentes *et al.* 1994) and a derivative reporter strain (T11-76), made by transformation of T1P11 with a reporter construct, had high levels of *aod-1* transcript following growth in non-inducing conditions compared to standard wild-type lab strains. Despite the presence of this mRNA, no AOD1 protein was detected in the mitochondria of T11-76 under non-inducing conditions. These observations raised two questions. Why were relatively high levels of *aod-1* mRNA produced in the absence of inducing conditions in this strain and; why was no AOD1 protein observed in the strain as a result of the high levels of mRNA?

Previously, analysis of a cross between T1P11 and a strain (NCN233) with a wild type tyrosinase gene, and the typically low levels of uninduced *aod-1* mRNA, showed that the high uninduced *aod-1* transcript levels in T1P11 did not segregate with the mutant allele of the tyrosinase gene (Descheneau *et al.* 2005). However, following this previous analysis, the progeny of this original cross were lost. To verify the previous data, and to generate strains for further analysis, we repeated the cross and analyzed 11 progeny strains. The presence of the *T*^-^ or *T^+^* allele was evaluated in the progeny by PCR amplification and sequencing of the region of the *T* gene known to contain mutations induced by repeat induced point mutation (Selker 1990) in the *T*^-^ allele (Descheneau *et al.* 2005). Progeny strains (referred to by their isolation numbers) 3, 4, 19, 34, and 36 were wild-type (*T^+^*) for the tyrosinase gene, while strains 6, 9, 16, 23, 26, and 33 carried the mutated (*T*^-^) allele of the tyrosinase gene (Table 1).

**Figure 1:**
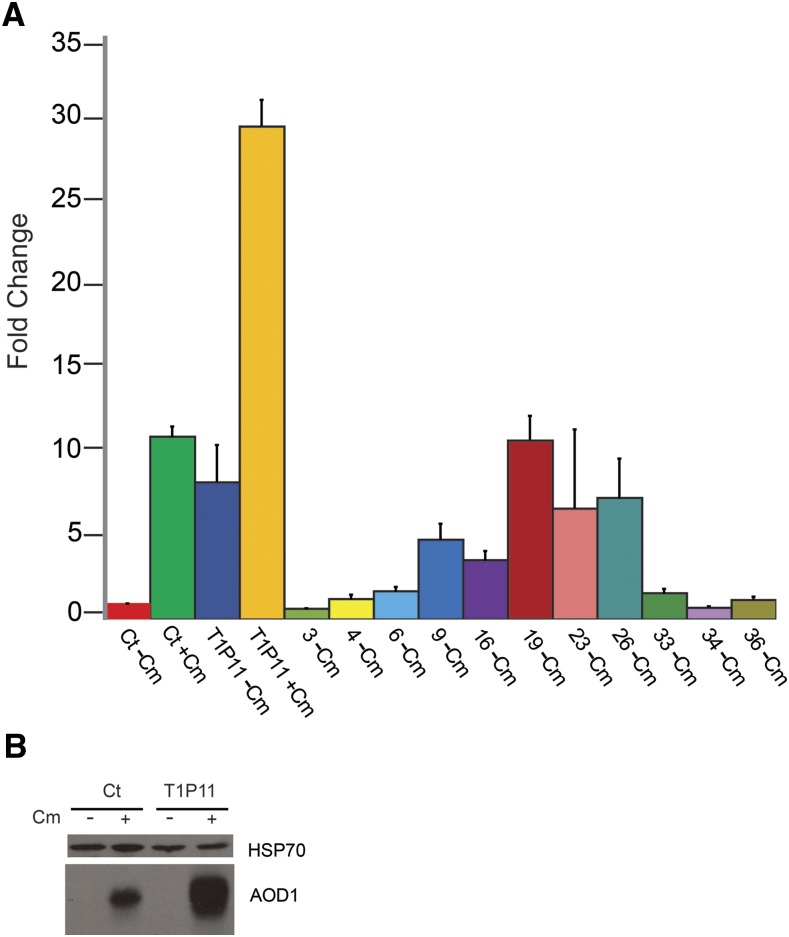
*aod-1* transcript levels in the standard laboratory strain NCN233 as a control (ct), T1P11, and progeny from a cross of the two strains indicated by the strain isolation number (Table 1). A, Strains were grown in medium lacking Cm (-Cm) for 16 hr or for 18 hr in medium containing Cm (+Cm). RNA was extracted and cDNA was generated. The expression levels of *aod-1* mRNA were determined by qPCR with four biological replicates (each with three technical replicates) of each strain and growth condition, standardized to β-tubulin. Levels are shown as the fold change relative to the control strain grown in the absence of Cm set as one. Error bars represent the standard error of the mean. B, AOD1 protein levels in parental strains. Mitochondria were isolated from NCN233 (Ct) and T1P11 following growth in both inducing (+Cm) and non-inducing (-Cm) conditions. Samples containing 30 μg of mitochondrial protein were subjected to SDS-PAGE and then blotted to nitrocellulose. The blot was probed with antibody to the AOD1 protein. Antibody to mitochondrial HSP70 was used as a loading control.

We then determined the levels of *aod-1* mRNA present in the parental and progeny strains by qPCR. As shown in Figure 1A, the control strain contained roughly ten-fold the amount of *aod*-1 mRNA when grown in inducing conditions (+Cm) relative to non-inducing (-Cm) conditions. However, uninduced cultures of strain T1P11 contained levels of mRNA approaching the induced level in control cells. Furthermore, induced T1P11 cultures had about three times more *aod-1* mRNA than induced control cells. Examination of AOD1 protein levels in the parental strains confirmed that no AOD1 protein could be detected in uninduced T1P11, despite the high levels of *aod-1* mRNA in these cells (Figure 1B). We also showed that the increased level of mRNA seen in induced T1P11 cells gave rise to more AOD1 protein than found in induced control cells (Figure 1B). For the progeny strains there was a range of uninduced mRNA expression levels. We arbitrarily defined progeny that had greater than 3.5 times the level of *aod-1* mRNA present in uninduced control cells as having “high” expression (progeny strains 9, 16, 19, 23 and 26), while progeny with below 3.5 times the level of uninduced control cell mRNA were designated as having “low” expression (progeny strains 3, 4, 6, 33, 34, 36). With respect to the traits of tyrosinase (*T^+^*or *T*^-^) and *aod-1* mRNA levels (high or low), the cross yielded four parental, high *aod-1* mRNA, *T*^-^ progeny (9, 16, 23 and 26) and four parental, low *aod-1* mRNA *T^+^* progeny (3, 4, 34, and 36). However, recombinant types were also recovered. Strain 19, which is *T^+^*, had elevated levels of *aod-1* transcript, while strains 6 and 33, which are *T*^-^, had low levels of *aod-1* transcript (Figure 1, Table 1). These data agree with the previous conclusion that the state of the tyrosinase gene is not responsible for the uninduced high level of expression of *aod-1* that is observed in some strains (Descheneau *et al.* 2005). In addition, although the number of progeny analyzed was low, the finding that six progeny had low levels of *aod-1* transcripts, while five had high levels, suggested that the expression level phenotype is determined by a single gene. However, given the arbitrary designation of high and low expression, and the low number of progeny examined, we cannot exclude the possibility that more than one gene may contribute to uninduced *aod-1* expression.

### Identifying the gene responsible for high uninduced aod-1 mRNA expression

The most obvious explanations for the production of *aod*-1 mRNA in strain T1P11 in non-inducing conditions would include a mutation in the *aod-1* promoter that allows it to be transcribed in the absence of an inducing signal, or a mutation in either the *aod-2* or *aod-5* genes that would make these known transcription factors for *aod-1* constitutively active. Since the transcription factors are considered to be constitutively bound to the promoter of the genes they regulate (Qi *et al.* 2017) a change in the amino acid sequence of AOD2 or AOD5 could be responsible for the production of *aod-1* mRNA seen in T1P11. In this regard, it should be noted that mutations affecting the RSE2 and RSE3 proteins of the fungus *Podospora anserina* (the orthologs of *N. crassa*’s AOD2 and AOD5, respectively), which result in higher levels of *P. anserina* alternative oxidase transcripts in non-inducing conditions, have been identified (Sellem *et al.* 2009).

To address these possibilities, we sequenced the promoter region of *aod-1*, and the genes for the transcription factors *aod-2* and *aod-5* in strain T1P11. The sequences obtained completely matched those of the reference sequence with one exception. There was a single transition (T to C) in the 5′ untranslated region (UTR) of *aod-5*. The change occurs at position -815 relative to the A of the ATG start codon taken as position +1. It seems very unlikely that a change in the 5′UTR of *aod-5* could cause the protein to become constitutively active. Thus, it appears that there are no relevant changes in the T1P11 sequence at the *aod-1*, *aod-2* or *aod-5* loci that would account for the observed increase in transcripts.

### Whole genome sequencing of high and low aod-1 transcript expressing strains

In an attempt to identify other genes that might be responsible for the upregulation of *aod-1* transcripts in non-inducing conditions, genomic DNA was extracted from several progeny and the parental strains of the NCN233 x T1P11 cross. Strains 3, 4, and 6, were chosen as low expressing progeny while 19, 23, and 26 were chosen as high expressing (Figure 1, Table 1). The sequenced DNAs were mounted in g-browse (http://ascobase.cgrb.oregonstate.edu/cgi-bin/gb2/gbrowse/ncrassa_public/) and a “.vcf” file was generated containing the data for all strains. This file was exported to Microsoft Excel, and analyzed (see Materials and Methods) to find changes common to the high expressing strains that were not present in the low expressing strains.

**Figure 2:**
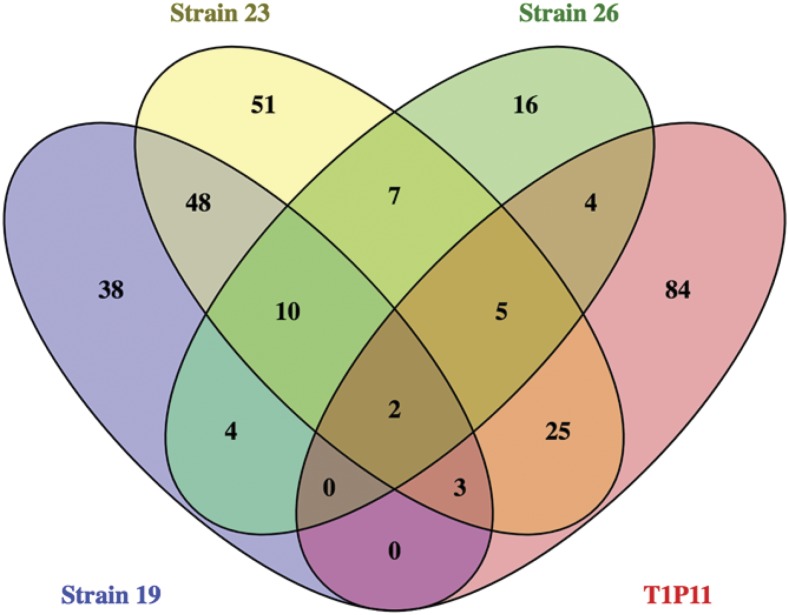
Venn diagram of changes, relative to the reference sequence, in coding regions of genes found to be altered in each of the four strains expressing high levels of uninduced *aod-1* transcript. Positions of changes in coding sequences found only in high-expressing strains were inserted into the FindData VBA program and individual NCU numbers were identified for each strain (see Materials and Methods). NCU numbers found to be in common among the strains were determined by the VENNY program (Oliveros 2007-2015) which displays the data in a Venn diagram. The strains used in the comparison are given beside the appropriate color coded oval. The number “2” in the center of the diagram represents the two genes *kin-9* (NCU05180) and *flbA* (NCU08319) that contained changes common to all four strains.

A large number of changes among the high expressing strains were identified when the whole genome sequences were considered (Table 2). We then examined the Excel sequence files for all differences within exons of protein coding regions of genes in the high expressing strains (T1P11, 19, 23, and 26) relative to the OR74A assembly 10 reference sequence transcript annotations. Only those changes that were not present in any low expressing strains were considered further (Table 2). These comparisons revealed 123 protein coding genes that were changed in T1P11, 105 genes in strain 19, 151 genes in strain 23, and 48 genes in strain 26. The genes in these four strains were then compared using a Venn diagram (Oliveros 2007-2015) to identify any changes common to all four strains (Figure 2). This analysis revealed mutations in two candidate genes, *kin-9* (NCU05180) and *flbA* (NCU08319). The complete sequence of these two genes was then further compared between the reference sequence and the high expressing strains.

**Table 2:** DNA sequence changes in strains expressing high levels of *aod-1* mRNA

Strain	T1P11	19	23	26
**Changes relative to the OR74A assembly 10 reference sequence**	19166	55628	62011	29164
**Changes after excluding those present in low expressing strains**	4434	1932	2842	2932
**Changes in protein coding regions after excluding those present in low expressing strains**	123	105	151	48

**Figure 3:**
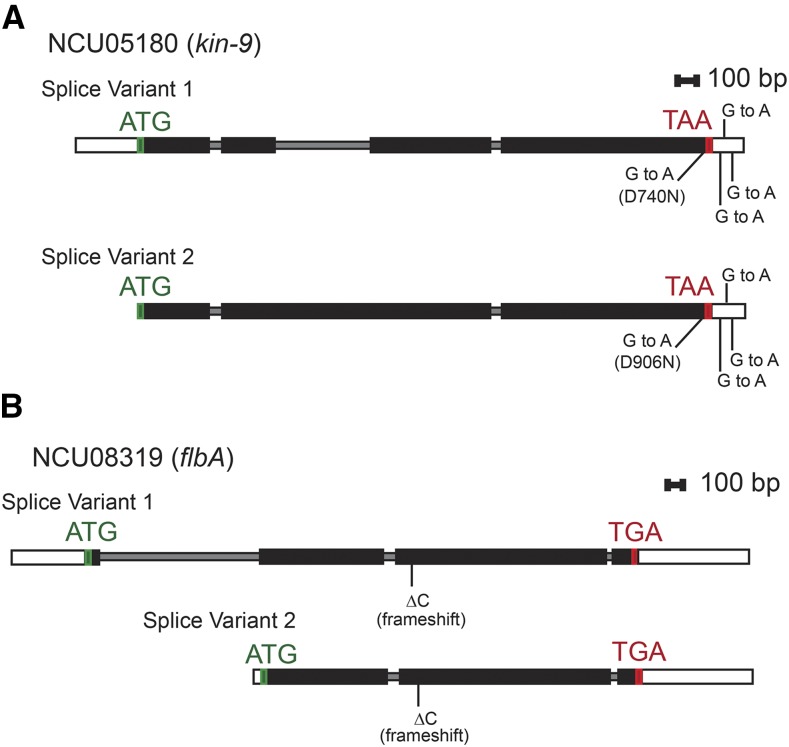
Location of mutations in *kin-9* (NCU05180) and *flbA* (NCU08319) in strains T1P11, 19, 23 and 26. Strains that had high uninduced *aod-1* expression levels (T1P11, 19, 23, 26) carry two mutated genes in common to all four strains. White rectangles represent UTRs, smaller gray rectangles represent introns, and black rectangles represent exons. The translational start codons (ATG) are indicated in green and stop codons (TAA or TGA) are shown in red. A, The *kin*-9 gene (NCU05180). A transition mutation (G to A) was located in codon 740 of splice variant 1, or in codon 906 of splice variant 2 resulting in an amino acid change from aspartic acid (Asp) to asparagine (Asn) in exon 3, or 4, depending on the splice variant. Three other G to A transitions, indicated by vertical lines above and below the 3′UTR, were located 3, 176, and 179 bp after the stop (TAA) in the 3′UTR. B, The *flbA* gene (NCU08319). A deletion mutation was located at codon 338 of splice variant 1, or codon 322 of splice variant 2 resulting in a frameshift in exon two or three that gives rise to a stop codon that would truncate the protein from 766 amino acids to 375 amino acids in variant 1, or 750 amino acids to 359 amino acids in variant 2.

**Figure 4:**
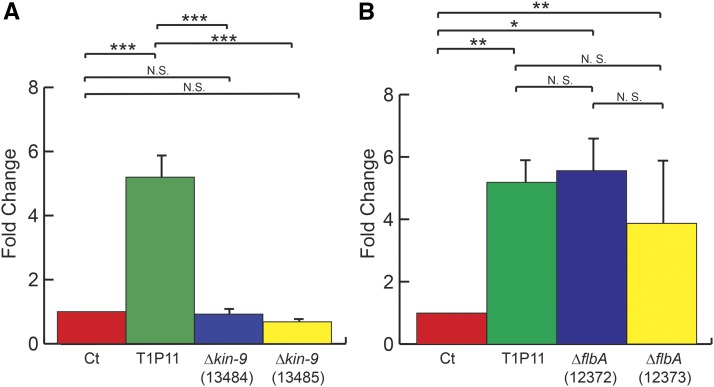
*aod-1* transcript levels in the *Δkin-9* and *ΔflbA* strains. A, *aod-1* transcript levels in *Δkin-9* knockout strains. Strains were grown in non-inducing conditions for 14 hr, RNA was extracted and cDNA was generated. The expression levels of *aod-1* mRNA were determined by qPCR with four biological replicates (three technical replicates each) and standardized to β-tubulin. ΔΔCT values were obtained by standardizing to the control (Ct) NCN233. Levels for the high expressing strain T1P11 are also shown for comparison. Error bars represent the standard error of the mean. The FGSC numbers for the deletion strains are given below the gene name. B, as in panel A but for the *ΔflbA* deletion strains. N.S., not significant; *, significant difference *P* = 0.05; **, significant difference *P* = 0.01; ***, significant difference *P* = 0.001.

The *kin-9* gene in all high expression strains contained four changes relative to the reference sequence (Figure 3A). All four changes were present in the *kin-9* allele of the T1P11 parental strain of the cross and the progeny that inherited that allele. Three of these were single nucleotide G to A changes in the 3′UTR. The fourth was also a G to A transition that occurred in the coding region of exon 3 or 4, depending on the splice variant considered. The latter transition would result in an amino acid change from aspartic acid to asparagine at residue 740 of the 743 residue protein, or 906 of the 909 residue protein, depending on the splice variant. The amino acid sequences of *kin-9* orthologs from five fungal species in the phylum Ascomycota were aligned. The Asp residue was conserved in species more closely related to *N. crassa* such as *Magnaporthe oryzae*, and *Aspergillus nidulans*, but was not conserved in the more distant species *Schizosaccharomyces pombe* and *Yarrowia lipolytica* (Figure S2). AmiGO2 gene ontology bioinformatics analysis (Ruepp *et al.* 2004) revealed that the gene product of *kin-9* is most likely involved with the cytoskeleton and is predicted to be associated with microtubules and vesicle mediated transport. Examining the NCU05180 predicted protein sequence using the Interpro protein sequence analysis and classification tool (McWilliam *et al.* 2013) revealed a conserved kinesin motor domain that was present in all five of the compared species (Figure S2). Given the predicted role for the NCU05180 protein, the fact that the amino acid change observed in NCU05180 of the *aod-1* high expressing strains is relatively conservative, and that the change does not occur in the functional domain identified in the protein, it seemed unlikely that this mutation would have an effect on *aod-1* transcript levels. This was confirmed by qPCR analysis of strains carrying a deletion of the *kin-9* gene found in the *N. crassa* knockout library (Colot *et al.* 2006). No significant difference in *aod-1* mRNA levels was seen in the strains compared to the control (Figure 4A).

The second change common to the four high expressing strains and absent in all the low expressing strains occurred in the gene encoding the NCU08319 protein, which is an ortholog of the *A. nidulans* FlbA protein (Figure S3). FlbA is an RGS protein that interacts with the Gα protein of heterotrimeric G proteins. G protein signaling is known to play a role in a number of cellular processes in a large number of organisms (Neves *et al.* 2002; Li *et al.* 2007; Syrovatkina *et al.* 2016; Pandey and Vijayakumar 2018). Briefly, the typical signaling cascade consists of a heterotrimeric G protein complex that contains G_α_, G_β_, and G_γ_ subunits, which exists in an inactive state associated with a GPCR (G protein coupled receptor) that is localized to the cell membrane. In this inactive state, GDP is bound to the G_α_ subunit. Upon interaction of the GPCR with an extracellular ligand, GDP is exchanged for GTP on the G_α_ subunit. This results in the dissociation of the heterotrimer from the GPCR and the further dissociation of G_α_ from G_β_G_γ_. In their dissociated forms, both G_α_ and G_β_G_γ_, are able to regulate downstream effectors. Eventually, a GTPase activity carried on the G_α_ subunit converts the bound GTP to GDP and restores G_α_ to its inactive form, which also enables rebinding and inactivation of G_β_G_γ_. RGS proteins interact with the G_α_ subunit and accelerate its native GTPase activity, thus reducing the time that G protein signaling would be active. Both the *N. crassa* and *A. nidulans* FLBA proteins contain an RGS domain as well as two DEP (disheveled, EGL-10, pleckstrin) domains (Figure S3) (Wang *et al.* 2013). The RGS domain is required for interaction with G_α_ proteins (Ross and Wilkie 2000; Siderovski and Willard 2005; Soundararajan *et al.* 2008; Kosloff *et al.* 2011). DEP domains have been shown to promote association of proteins with the cell membrane, but may also promote association with specific GPCRs so that a given RGS protein might be localized to sites that contain its substrate G_α_ protein (Ballon *et al.* 2006; Chen and Hamm 2006; Consonni *et al.* 2014).

The change in common to the four overexpressing strains in the *flbA* gene was a single C-G base pair deletion in exon 2 (codon 320) or exon 3 (codon 336), depending on the splice variant of the gene (Figure 3B). None of the strains expressing low levels of *aod-1* transcript contained the mutation. The deletion would result in a frameshift in the coding sequence leading to the creation of a stop codon and truncation of the encoded protein from 766 amino acids to 375 amino acids for splice variant 1, or 750 amino acids to 359 amino acids for variant 2. This mutation would have a drastic effect on the function of the protein given that both the RGS and DEP domains would be lost (Figure S3). Since the normal function of RGS proteins is to accelerate the GTPase activity found in the Gα subunit of heterotrimeric G proteins, inactivation of the RGS protein would result in prolonged activity of the active GTP bound form of Gα.

### Data supporting the effect of the flbA mutation on aod-1 transcript levels

The Neurospora genome project (Colot *et al.* 2006) has reported an abnormal morphology at the edge of the mycelium of *flbA* deletion strains growing on plates at 37° (http://fungidb.org/fungidb/). Under these conditions of growth, we found that the growing mycelium of the strains expressing low levels of *aod-1* transcript had a diffuse and uneven edge, while the high expressing *flbA* mutant strains had a compact and even edge, that was similar to that of the deletion strains (Figure S4).

Various studies have been conducted on *flbA* mutants of *Aspergillus* species. Directly relevant to our study is a result from a microarray analysis on an *A. niger ΔflbA* strain which found that one of the top 50 upregulated genes in the *ΔflbA* background was the *A. niger* AOX-encoding gene, *aox1* (Krijgsheld and Wösten 2013). This observation supports the hypothesis that the frameshift mutation found in the *flbA* gene in the high *aod-1* expressing strains of *N. crassa* is responsible for the upregulation. To further test this hypothesis, we examined strains lacking the *flbA* gene from the *N. crassa* single gene deletion library (FGSC strains 12372 and 12373, Table 1) for levels of *aod-1* transcript in non-inducing conditions and found elevated *aod-1* transcript levels as observed in T1P11 (Figure 4B). Taken together, these data strongly support the hypothesis that loss of function of the *flbA* gene is responsible for the high uninduced levels of *aod-1* mRNA in strain T1P11 and other high expressing strains.

**Figure 5:**
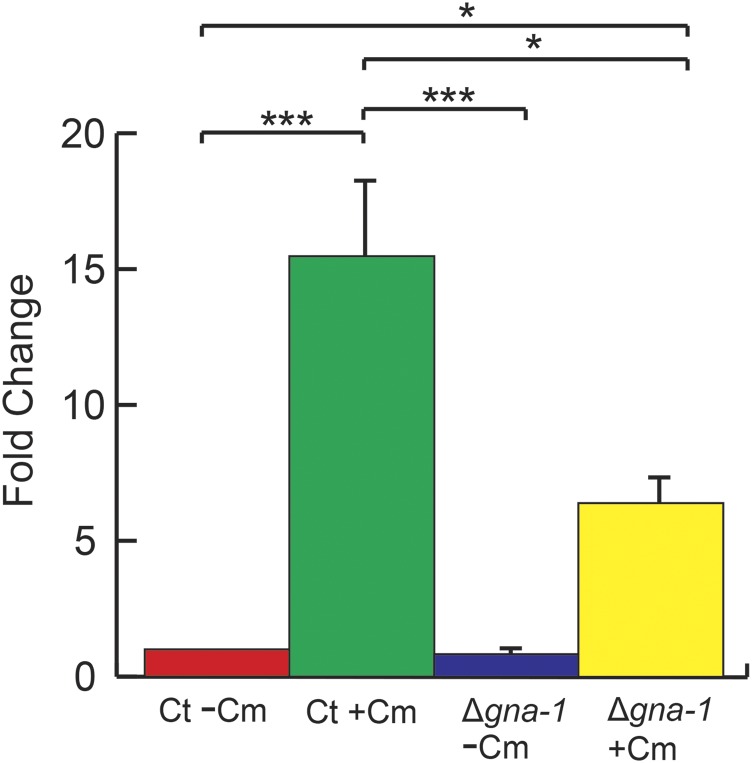
Levels of *aod-1* mRNA in the *Δgna-1* strain (FGSC #12370). Analysis was as described in the legend to Figure 4.

The results described above suggested that loss of the RGS protein encoded by the *flbA* gene resulted in a Gα protein that is active for a longer time, which would further lead to an increase in *aod-1* transcripts. Thus, it would be predicted that loss of the Gα protein controlled by the FLBA protein should lead to a reduction of *aod-1* expression. In *A. nidulans*, the FlbA protein interacts with the Gα protein FadA (Yu *et al.* 1996; Yu *et al.* 1999). The Neurospora ortholog of FadA is a class I fungal Gα protein called GNA1 (NCU06493) that is most closely related to the Gα_i_ family in mammals (Turner and Borkovich 1993; Ivey *et al.* 1996; Bölker 1998; Li *et al.* 2007). GNA1 is known to be involved in various aspects of growth and development in *N. crassa* (Kays and Borkovich 2004; Li *et al.* 2007). Interestingly, we previously identified the *gna-1* deletion strain in the *N. crassa* single gene deletion library, as having a slight deficiency of the AOD1 protein when grown in the presence of Cm (inducing conditions) (Nargang *et al.* 2012). To determine if levels of *aod-1* mRNA were also reduced in the *Δgna-1* strain, we performed qPCR analysis following growth in both inducing and non-inducing conditions. A reduction in *aod-1* mRNA levels of about three fold was seen in the *Δgna-1* strain grown under inducing conditions (+Cm) relative to the control (Figure 5), supporting the predicted role of the protein based on the effects seen in the *flbA* mutants. However, no significant reduction in *aod-1*transcript levels was detected in uninduced cultures (Figure 5). Thus, it seems likely that in the absence of an inducing signal, the presence or absence of GNA1 has little effect on *aod-1* transcription.

### Detectable AOD1 protein in some cultures of uninduced strain 23

**Figure 6:**
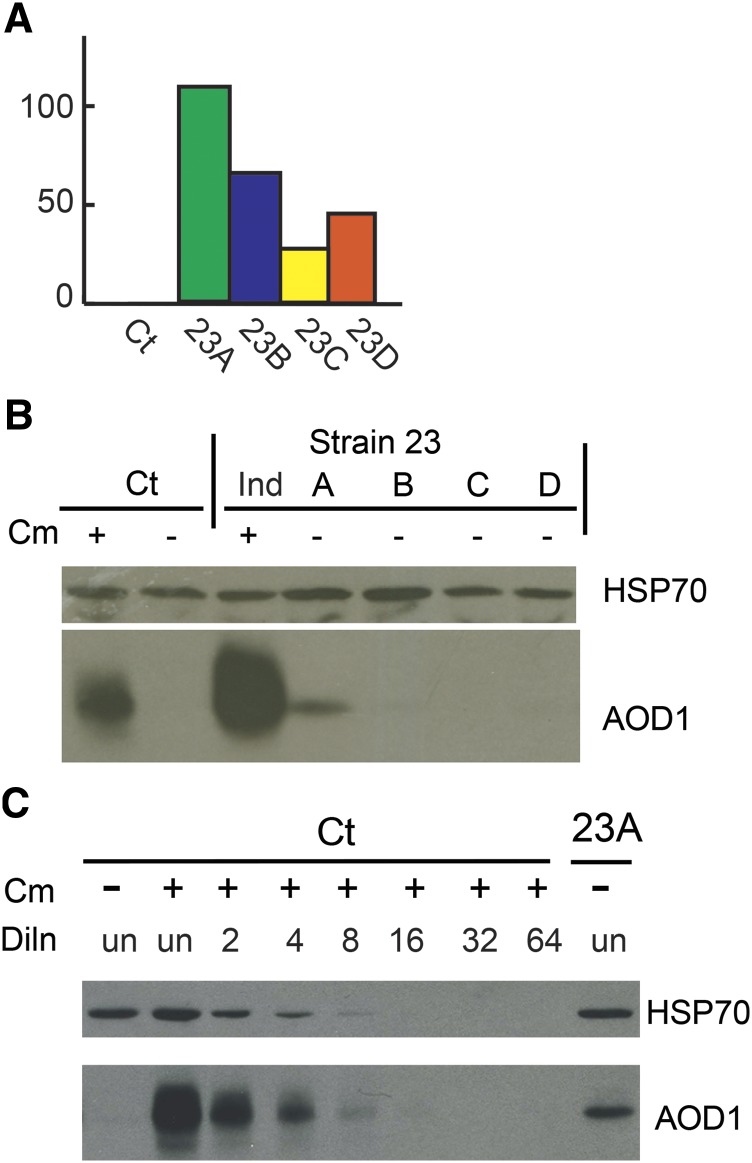
Levels of *aod-1* mRNA and AOD1 protein in four separate individual cultures (designated A, B, C, D) of strain 23. A, qPCR analysis of transcript levels. Analysis was as in the legend to Figure 4, except that each culture was a single biological replicate. B, AOD1 protein levels. Mitochondria were isolated from an induced (+Cm) and an uninduced (-Cm) culture of the control (Ct) strain NCN233, the four individual uninduced (-Cm) cultures of strain 23, and one induced (Ind) culture of strain 23. Samples (30 μg mitochondrial protein) from each strain were subjected to SDS-PAGE followed by blotting to nitrocellulose. The blot was probed with antibody to the AOD1 protein. Antibody to mitochondrial HSP70 was used as a loading control. C, Abundance of AOD1 protein in strain 23 uninduced culture A relative to AOD1 level in an induced control (Ct) culture, NCN233. An undiluted (un) sample of mitochondria containing 30 μg mitochondrial protein isolated from an induced (+Cm) control culture, and samples diluted (Diln) from 2 fold to 64 fold, were electrophoresed together with an undiluted (un) sample of mitochondria (30 μg mitochondrial protein) from the uninduced (-Cm) strain 23 culture A. An undiluted sample of uninduced (-Cm) NCN233 mitochondria containing 30 μg mitochondrial protein was included as a control.

Our measurements of *aod-1* mRNA levels in uninduced cultures revealed considerable variation among biological replicates of the high expressing strains, most notably for strain 23 (see error bars in Figure 1 for strains T1P11, 19, 23, and 26). This result raised a question about our earlier observation that strains with high levels of *aod-1* mRNA contained no AOD1 protein (Descheneau *et al.* 2005). That is, it was possible that protein levels in previous studies had been measured in cultures that may have contained, by chance, the lower levels of mRNA that sometimes occurs in these strains. To address this question, we grew several separate uninduced cultures of the high expressing strain 23 and isolated both RNA, for determination of *aod-1* mRNA levels; and mitochondria, for determination of AOD1 protein levels, from each culture. The proteins of mitochondria isolated from four of the cultures with very high *aod-1* mRNA levels (strain 23 cultures A, B, C, and D, Figure 6A), were examined on western blots for the presence of the AOD1 protein (Figure 6A). As seen in our previous results, the mitochondria from three of these cultures contained no detectable AOD1 protein, despite having levels of *aod-1* mRNA higher than in induced wild type cells (compare Figure 6A and Figure 1). However, strain 23 culture A was found to contain detectable levels of AOD1 protein, though at lower levels than induced control cells (Figure 6B). Of the four strain 23 cultures examined, culture A contained the highest level of *aod-1* mRNA. The levels were about 100-fold higher than the uninduced control (Figure 6A) and about ten times higher than in induced control cells (compare Figure 6A and Figure 1).

To estimate the level of AOD1 protein found in strain 23 culture A, relative to the amount present in an induced wild type control, twofold serial dilutions of mitochondria isolated from a +Cm induced culture of the control NCN233 strain were electrophoresed on a gel that also contained a mitochondrial sample from culture A of strain 23. The gel was then examined by western blot using antibody to the AOD1 protein. Comparison of the band intensities revealed that the protein abundance for replicate A grown in the absence of Cm was approximately fourfold less than the protein amount of NCN233 grown in the presence of Cm (Figure 6C). However, the amount of *aod-1* transcript in NCN233 +Cm cultures was typically about 10-fold more than in uninduced NCN233 cultures (Figure 1), while the strain 23 replicate A had 100 times as much transcript as uninduced NCN233 (Figure 6A). Based on these numbers for the transcripts it would be predicted that replicate A would have approximately 10-fold more AOD1 protein than induced NCN233 rather than fourfold less. This finding, coupled with the observation that most uninduced cultures with high levels of *aod-1* mRNA have no detectable AOD1 protein, strongly supports the hypothesis that a mechanism of post-transcriptional control, most likely at the level of translation, exists for *aod-1* mRNA in non-induced cultures.

## Discussion

The *N. crassa aod-1* gene encodes an AOX, called AOD1, which is able to transfer electrons directly from ubiquinol to molecular oxygen. Standard lab strains of *N. crassa* contain very low levels of *aod-1* mRNA when grown under non-inducing conditions (Tanton *et al.* 2003; Chae *et al.* 2007b; Qi *et al.* 2017). These levels increase several fold when cells are grown in inducing conditions, such as in the presence of inhibitors that decrease the function of the standard electron transport chain, or if cells contain mutations affecting the function of the standard chain. Here, we have addressed the previous observation that strain T1P11 contained high levels of *aod-1* transcript even in non-inducing conditions (Descheneau *et al.* 2005).

Analysis of whole genome sequences of T1P11, a standard laboratory strain expressing low levels of *aod-1* transcript in non-inducing conditions (NCN233), and six progeny derived from a cross of these two strains revealed mutations in two genes, *kin-9* (NCU05180) and *flbA* (NCU08319), that might be responsible for the phenotype. Both of these mutations were present in all high expressing strains and no low expressing strains. Further analysis provided no evidence supporting the involvement of the *kin-9* gene mutations in the phenotype. However, several observations supported the hypothesis that the frameshift mutation identified in the *flbA* gene is responsible for the high level of *aod-1* transcripts under non-inducing conditions. First, the frameshift occurs early in the gene and would result in loss of the conserved functional domains that are predicted in the protein. Second, levels of *aod-1* mRNA were also found to be high in the *ΔflbA* strains from the *N. crassa* single gene deletion library when grown in non-inducing conditions. Third, based on the function of both the RGS protein encoded by *flbA* and the Gα protein GNA1, with which it interacts, we observed the predicted decrease in *aod-1* mRNA in a strain carrying a deletion of the *gna-1* gene when grown under inducing conditions. Finally, a transcriptome study in an *A. niger* designed to assess the affects of a deletion of *flbA* on spatial gene expression found that transcripts from the AOX encoding gene *aox-1* were upregulated (Krijgsheld and Wösten 2013).

To our knowledge there have been no detailed studies on the role of the FLBA protein in *N. crassa*. However, initial analysis of *flbA* deletion mutants as part of the *N. crassa* genome project revealed that the strains had an abnormal growth pattern on plates at 37°, the morphology of protoperithecia was altered, and mature perithecia were not formed (http://fungidb.org/). The role of the *N. crassa* gene has also been considered as part of a network required for perithecial development in filamentous fungi in an evolutionary context (Trail *et al.* 2017). We have confirmed that the *flbA* frameshift identified in our study results in a growth phenotype similar to the deletion strains (Figure S4).

The function of the *flbA* gene has been studied more extensively in the *Aspergillus* species *A. niger*, *A. nidulans*, and *A. fumigatus*. Deletion *flbA* strains were found to have uncontrolled growth, inability to form conidiophores, and to accumulate undifferentiated aerial hyphae that are subject to autolysis (Lee and Adams 1994; Wieser *et al.* 1994; Yu *et al.* 1996). Changes in the production of secondary metabolites (Hicks *et al.* 1997; Aerts *et al.* 2018) and in the secretome (Krijgsheld *et al.* 2013) have also been described. The transcriptome study that revealed the up regulation of AOX transcripts in the *flbA* mutant also identified twofold or greater changes in expression of 1152 different genes, including 38 that were predicted to encode transcription factors (Krijgsheld and Wösten 2013). However, the *A. nidulans* orthologs of AOD2 and AOD5 were not among the transcription factors with altered expression.

Deletion of *flbA* in *A. fumigatus* has been found to have effects on a number of processes, including increased expression of the GliT protein (Shin and Yu 2013; Shin *et al.* 2013), which is required for both synthesis and self resistance to gliotoxin (Schrettl *et al.* 2010). In addition, an increase in expression of catalase and an increase in intracellular activity of superoxide dismutase (SOD) were noted. These proteins play a role in coping with reactive oxygen species (ROS). Furthermore, an *A. fumigatus* strain expressing a constitutively active GpaA (the ortholog of the *A. nidulans* Gα protein FadA), also had higher expression of the catalase encoding genes *cat1* and *cat2* (Shin and Yu 2013), supporting the notion that the effects seen in the *flbA* deletion strain are mediated via the G protein signaling system. These observations led to the proposal that the FlbA protein and a G protein regulatory circuit are involved in signaling that affects the cell’s response to reactive oxygen species (ROS) (Shin and Yu 2013). Interestingly, AOX has also been shown to have a role in responding to ROS in many fungi, especially in pathogenic species where ROS are generated as a defense mechanism by the host (Yukioka *et al.* 1998; Akhter *et al.* 2003; Magnani *et al.* 2008; Ruiz *et al.* 2011; Cárdenas-Monroy *et al.* 2017). Conceivably, AOX may be another factor in an overall G protein regulatory circuit that responds to ROS in such fungi. However, it seems unlikely that ROS are involved in activating a similar system in *N. crassa*, since strains expressing constitutively active GNA1 protein have increased sensitivity to oxidative stress (Yang and Borkovich 1999).

Our findings that the knockout of *gna-1* has decreased levels of *aod-1* mRNA in inducing conditions, while the *flbA* mutation gives rise to increased expression in non-inducing conditions, argues that a classic G protein signaling pathway plays a role in *aod-1* expression in *N. crassa*. This further implies that an extracellular signal may be influencing *aod-1* expression, since the signaling pathway should be induced by an extracellular ligand binding to a GPCR. We do not know the nature of this signal, nor do we know the downstream effector that is influenced by the active GNA1 Gα protein. The most likely possibilities for the latter may be cascades involving either cAMP or mitogen activated protein kinases (MAPK) (Li *et al.* 2007; Won *et al.* 2012).

One gene identified in an *A. fumigatus flbA* deletion study suggests a link to the expression of AOX encoding genes in various fungi. The *acuF* gene, which encodes phophoenolpyruvate carboxykinase (PEPCK), a key gluconeogenic enzyme, was found to be upregulated in the *flbA* deletion strain (Shin *et al.* 2013). In *A. nidulans*, expression of *acuF* is known to require the transcription factors AcuK and AcuM, which are also required for transcription of the AOX encoding gene (*aodA*) (Suzuki *et al.* 2012). AcuK and AcuM are the orthologs of *N. crassa* AOD2 and AOD5, which are required for transcription of both *aod-1* and the gene encoding PEPCK (*acu-6*) in *N. crassa* (Chae *et al.* 2007b; Qi *et al.* 2017). Similarly, expression of the genes encoding PEPCK and AOX in *Podospora anserina* requires the orthologs of AOD2 and AOD5 (Bovier *et al.* 2014). Thus, it is conceivable that one of the effects mediated by the active Gα protein results is the activation of the AOD2 and AOD5 transcription factors (or their orthologs).

Is the G protein signaling system entirely responsible for the upregulation of *aod-1* transcription in inducing conditions? Although our measurements of *aod-1* transcript levels, especially in highly expressing strains, were subject to variation, we generally observed less *aod-1* mRNA in uninduced *flbA* mutant strains than in induced wild type strains. In addition, more *aod-1* transcript was present when we examined *flbA* mutants grown under inducing conditions compared to the same strain in non-inducing condtions (Figure 1 and Descheneau *et al.* 2005). These observations could be interpreted as a second signaling system playing an additive role under inducing conditions, but could also reflect a fuller activation of the G protein system in inducing conditions. However, the observation that the *Δgna-1* strain, grown under inducing conditions, still contains significantly more *aod-1* transcript than uninduced wild type strains (Figure 5), argues for a second system of transcriptional control in addition to that using the Gα protein GNA1.

As stated above, we have noted variation in *aod-1* transcript levels in high expressing strains as shown by the range of values and larger error bars (Figure 1). One possible explanation for this observation relates to the spatial transcriptome study in the *ΔflbA* mutant strain of *A. niger* (Krijgsheld and Wösten 2013). Transcript levels were measured in three different concentric zones of colony growth on agar plates: zone 1 was the oldest, central-most zone, zone 3 was the intermediate zone, and zone 5 was the youngest, most peripheral zone. The level of upregulation of *aox1* varied between zones. In the central zone, the expression of *aox1* was increased about sixfold over wild-type, in the intermediate zone expression was almost ninefold higher and at the periphery of growth about 19 fold higher than wild type. The cultures from which RNA was extracted for our qPCR analysis of *aod-1* transcript levels, were liquid cultures. Typically, the total culture amounted to about 8 g wet weight when harvested, but only 100 mg from these cultures was used for RNA extraction. The larger culture would consist of both young hyphal regions at growing tips as well as older regions where growth had initiated. There was no distinction between such regions used for RNA extraction. If a pattern of expression relating to age of the mycelium exists in *N. crassa*, as it does in *A. niger*, then simple random sampling may have played a role in generating the variability due to different ages of the mycelia that were taken.

We previously noted that the higher level of *aod-1* transcripts in uninduced cultures of strain T1P11 do not give rise to detectable AOD1 protein (Descheneau *et al.* 2005). Here we have shown that this is usually the case, but such cultures may occasionally contain low levels of AOD1 protein. However, even in such cases, the levels of protein detected were much lower than the amount predicted based on transcript levels when compared to induced wild type cells. This argues for the existence of a post-transcriptional control mechanism that responds to inducing conditions to allow translation of the message. Since the G protein signal resulting in increased transcription of *aod-1* under non-inducing conditions in *flbA* mutants does not give rise to efficient translation of the message, this implies that an additional signal, produced under inducing conditions, is required for translation.

**Figure 7:**
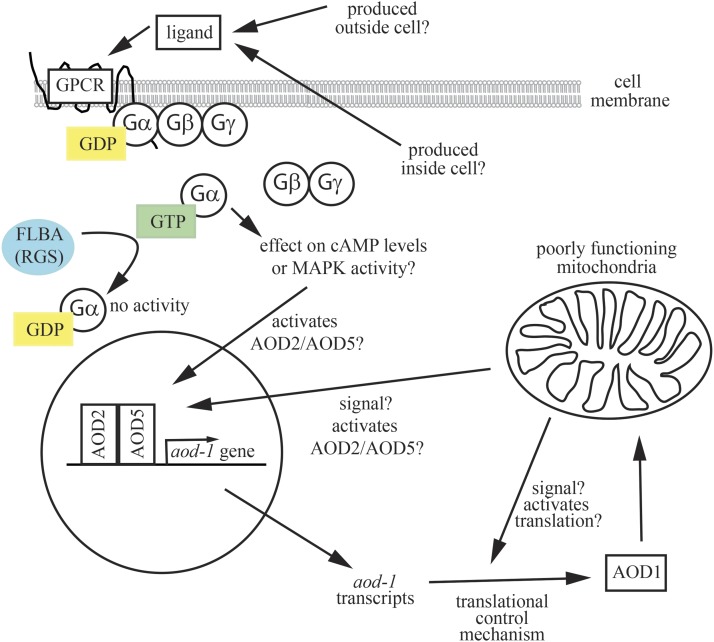
Summary of control of AOX production in *N. crassa*. The heterotrimeric GαGβGγ protein is shown bound to the membrane spanning GPCR. GDP (yellow) is bound to the Gα subunit. Upon binding an unknown ligand, which could be formed in the growth medium or secreted from inside the cell during inducing conditions, GDP is exchanged for GTP (green) and Gα dissociates from the membrane and from GβGγ. In this active state Gα acts on a downstream effector, possibly giving rise to increased cAMP levels or MAPK activity. Either of these might serve to activate the AOD2/AOD5 transcription factors. In normal cells grown under non-inducing conditions, the activity of Gα would be short lived, as the RGS protein FLBA (blue) would stimulate the GTPase activity of Gα and restore it to the inactive GDP bound state. However, in the *flbA* mutant strains, Gα activity would persist for much longer times. A separate signal(s) arising from poorly functioning mitochondria may also directly or indirectly activate transcription via AOD/AOD5. Similarly, a signal(s) produced under inducing conditions results in the translation of *aod-1* mRNA into the AOX AOD1 protein, which is then translocated into mitochondria.

In summary, our results support the view that there are at least three systems of control for production of the AOD1 AOX protein in *N. crassa* (Figure 7). Two systems appear to activate *aod-1* transcription. One is a G protein regulatory circuit that responds to an unknown signal produced when the function of the standard electron transport chain in mitochondria is compromised. Since the *gna-1* deletion strain grown under inducing conditions contains more *aod-1* transcript than uninduced wild type strains, this implies the existence of a second transcriptional regulatory system that also responds to a signal produced when the standard electron transport chain is compromised. Finally, since the excess *aod-1* mRNA produced under non-inducing conditions in mutant *flbA* strains is not efficiently translated, it appears there is a system for activation of translation of *aod-1* mRNA, which is also generated when the standard electron transport chain is compromised. Further investigation will be aimed at the nature of the signaling molecules and the interplay between the different systems of control.
